# Remodeling of the periodontal ligament and alveolar bone during axial tooth movement in mice with type 1 diabetes

**DOI:** 10.3389/fendo.2023.1098702

**Published:** 2023-01-23

**Authors:** Wenjin Li, Jing Zheng, Yao Xu, Weiran Niu, Dong Guo, Jianing Cui, Wenjin Bian, Xiaohui Wang, Jinliang Niu

**Affiliations:** ^1^ Department of Stomatology, 2nd Hospital, Shanxi Medical University, Taiyuan, Shanxi, China; ^2^ School of Basic Medicine, Shanxi Medical University, Taiyuan, Shanxi, China; ^3^ Stomatological Hospital of Shanxi Medical University, Taiyuan, Shanxi, China; ^4^ Department of Mental Health, Shanxi Medical University, Taiyuan, Shanxi, China; ^5^ Medical Imaging Department of Shanxi Medical University, Taiyuan, Shanxi, China; ^6^ Department of Radiology, 2nd Hospital, Shanxi Medical University, Taiyuan, Shanxi, China

**Keywords:** tooth movement, periodontal ligament, type 1 diabetes, alveolar bone remodeling, osteogenesis

## Abstract

**Objectives:**

To observe the elongation of the axial tooth movement in the unopposed rodent molar model with type 1 diabetes mellitus and explore the pathological changes of periodontal ligament and alveolar bone, and their correlation with tooth axial movement.

**Methods:**

The 80 C57BL/6J mice were randomly divided into the streptozotocin(STZ)-injected group (n = 50) and the control group (n = 30). Mice in the streptozotocin(STZ)-injected group were injected intraperitoneal with streptozotocin (STZ), and mice in the control group were given intraperitoneal injection of equal doses of sodium citrate buffer. Thirty mice were randomly selected from the successful models as the T1DM group. The right maxillary molar teeth of mice were extracted under anesthesia, and allowed mandibular molars to super-erupt. Mice were sacrificed at 0, 3, 6,9, and 12 days. Tooth elongation and bone mineral density (BMD) were evaluated by micro-CT analysis(0,and 12 days mice). Conventional HE staining, Masson staining and TRAP staining were used to observe the changes in periodontal tissue(0, 3, 6, 9, and 12 days mice). The expression differences of SPARC, FGF9, BMP4, NOGGIN, and type I collagen were analyzed by RT-qPCR.

**Results:**

After 12 days of tooth extraction, our data showed significant super-eruption of mandibular mouse molars of the two groups. The amount of molar super-eruption in the T1DM group was 0.055mm( ± 0.014mm), and in the control group was 0.157( ± 0.017mm). The elongation of the T1DM mice was less than that of the control mice(*P*<0.001). It was observed that the osteoclasts and BMD increased gradually in both groups over time. Compared with the control group, the collagen arrangement was more disordered, the number of osteoclasts was higher (*P*<0.05), and the increase of bone mineral density was lower(2.180 ± 0.007g/cm^3^ vs. 2.204 ± 0.006g/cm^3^, *P*<0.001) in the T1DM group. The relative expression of SPARC, FGF9, BMP4, and type I collagen in the two groups increased with the extension of tooth extraction time while NOGGIN decreased. The relative expression of all of SPARC, FGF9, BMP4, and type I collagen in the T1DM group were significantly lower, and the expression of NOGGIN was higher than that in the control group (*P*<0.05).

**Conclusion:**

The axial tooth movement was inhibited in type 1 diabetic mice. The result may be associated with the changes of periodontal ligament osteoclastogenic effects and alveolar bone remodeling regulated by the extracellular matrix and osteogenesis-related factors.

## 1 Introduction

Tooth movement is a multi-factor process involving the motion of existing tissues and the formation of new tissues coordinated by a set of genetic events (1), commonly occurring in tooth eruption, tooth loss, adaptation to mastication, orthodontic tooth movement, etc. (2). The alveolar bone surrounding the teeth is constantly remodeled throughout life (3) and presents a high turnover rate during tooth movement, including bone resorption and bone formation. The periodontal ligament(PDL) anchors the tooth in the alveolar socket. Extrinsic and intrinsic stimuli trigger the response of periodontal ligament fibroblasts to induce periodontal tissue reorganization, which is the biological basis of tooth movement (4). The tooth movement process involves high levels of elaborate interactions between growth factors, transcription factors, and structural proteins (5). Establishing the dynamic relationship among tooth movement, alveolar bone remodeling, and periodontal ligament biological responses is of great significance for orthodontics.

At present, studies of tooth movement have focused on tissue remodeling of horizontal tooth movement caused by orthodontic force (6). The horizontal movement during orthodontic treatment is affected by the traction force of the orthodontic device, and the mechanical stress or traction generated during orthodontic tooth movement also can cause tissue damage and triggers an inflammatory response (7), which cannot reflect the intrinsic physiological mechanism of tooth movement. The study of tooth axial movement is mainly related to tooth eruption, which is defined as the axial movement of the tooth from its site of development in the alveolar bone to its functional position in the oral cavity (8). Therefore, the research on axial tooth movement is of great significance to the study of the physiological mechanism of tooth movement and tooth eruption. However, there were few empirical researches on it. The research had demonstrated that after removing the upper molars unilaterally, the lower molars would erupt continuously without an antagonist, which was used to study the axial tooth movement (9).

In addition, tooth movement is also affected by medication or systemic pathological factors. Diabetes mellitus(DM) is a group of clinical syndromes, which will result in diminished bone-mineral density and osteoporosis (10–12). The study showed that DM not only induces higher alveolar bone resorption but also enhances fibroblast inflammation and osteoclastogenic effects of periodontal ligament (13). Some results suggested that tooth movement in T2DM animals was higher than in healthy animals with adapted orthodontic appliances (14), while another study observed that the amount of orthodontic tooth movement in T2DM was not compromised (15). However, relatively few data are available for type 1 diabetes mellitus (T1DM) (16). Studies have found that children with T1DM with age younger than 11.5 years had teeth eruption acceleration, while children older than 11.5 years had delayed teeth eruption process (17). Type 1 T1DM is becoming more prevalent among young individuals worldwide who is also the main population needing orthodontic treatment. Compared with T2DM, T1DM affects bone more severely and *via* a pathophysiological mechanism dependent on a decrease in bone mineral density (BMD) (18).The cellular and molecular related to T1DM that may affect the elongation movement of teeth in the axial direction are still unclear, and it is necessary to investigate these mechanisms.

The present study was performed to visualize the process of axial tooth movement by using T1DM the model of unopposed rodent molar with type 1 diabetes mellitus, and investigate the related changes of the periodontal ligament biological responses and alveolar bone remodeling.

## 2 Material and methods

### 2.1 Animals

C57BL/6J mice(six weeks old, male, 19~25g) were purchased from the Animal Laboratory Center of Shanxi Medical University(Animal Permit NO: DW2022038) and kept in a standard lab housing with a 12 h light/dark cycle at a temperature of 22 ± 2°C and 30% - 50% humidity with diet and water ad libitum. Study protocols were approved by the Animal Ethics Committee of the Second Hospital of Shanxi Medical University.

### 2.2 Induction of T1DM model

Eighty mice were divided into control(n=30) and streptozotocin(STZ)-injected groups(n=50) randomly. The mice were fasted for 8h before STZ injection. Injected mice were intraperitoneally injected a single dose of 150mg/kg STZ (Sigma S-0130) dissolved in 0.1 M citrate buffer at pH 4.2. The control group received the same volume of 0.1 M citrate buffer. 72h after the STZ injection, mice with consistent blood glucose levels ≥11.1 mmol/L for three consecutive days were regarded as successful diabetic models (19). T1DM mice were fed a diet high in fat and sugar. Plasma glucose concentration was recorded during the experimental period. The model was considered to be unsuccessful if blood glucose returned to normal or the mice died during feeding.

### 2.3 Induction of un-opposed molar model

The T1DM group(n=30) and the control group(n=30) were anesthetized with Ketamine (100mg/kg) and Xylazine (5mg/kg). All three right maxillary molars were extracted under anesthesia. The mice were intraperitoneally injected with buprenorphine (0.05 mg/kg) to manage pain after the operation.

### 2.4 Mandible morphometric analyses by microcomputerized

Mice were sacrificed using an excess of isoflurane anesthesia at 0 and 12 days after surgery. The mandibular alveolar bone was harvested, and bone specimens were scanned with a micro-CT scanner equipped with a custom software package (Micro-CTCH-8306, Scanco Medical, Basserdorf, Switzerland). Specimens were scanned at 70 kVp and 114 μA at high resolution (37 μm). The images were processed by three-dimensional reconstruction software (μCT Evaluation Program v6.0, Scanco Medical). Taking the line of the outermost tangent point above the bilateral mandibular foramen of mice as the reference line, the distance between the two parallel lines at 12 day after tooth extraction was measured from the highest point of the right mandibular first molar as the parallel line with the reference line, and the distance between the two parallel lines at 0 days after tooth extraction was subtracted as the eruption rate. Each sample was analyzed by two different investigators independently. The analysis software Mimics Medical 17.0 was used to measure bone mineral density (BMD).

### 2.5 Tissue processing

Mice were sacrificed using an excess of isoflurane anesthesia at 0, 3, 6, 9 and 12 days after surgery. Collected mandibles were fixed in 10% neutral formalin for 24h followed by decalcification for 30~40 days with 10% EDTA. After decalcification and paraffin embedding, the samples were cut in 4 μm sagittal sections along the long axis of the molar teeth, and the sections were stained with hematoxylin and eosin (H&E) Masson and Tartrate-resistant acid phosphatase (TRAP) staining. The results were observed under a microscope(Olympus, Tokyo, Japan). Scanscope scanning system was used to scan the stained sections, and Spectrum software was used to analyze the sections. Five high-magnification fields (×200) were randomly selected from the sections of each group of the diabetes group and the control group to count the osteoclasts. The results was analyzed by two different investigators independently.

### 2.6 RNA extraction and real-time quantitative PCR

Total RNA was extracted from the mandibles of three mice (per group). The expression of SPARC, FGF9, BMP4, type I collagen, and NOGGIN gene expression in the periodontal tissues were assessed by real-time quantitative PCR. RNAs underwent RT-PCR using Sprint RT Complete kit (Clontech). Real-time quantitative PCR was performed using Taqman Fast Universal PCR Master Mix (Applied Biosystems) with DLUX fluorogenic primers. Reaction conditions were as follows: 30s at 95°C (one cycle), 5s at 95°C, and 30s at 60°C (40 cycles). PCR products were continuously monitored with an ABI PRISM 7900 detection system (Applied Biosystems). Primer sequences used for real-time PCR analysis are shown in **Table 1**. The CT value of each group was computed and processed according to 2^-ΔΔCt^, and the results were plotted by the software GraphPad Prism.

**Table 1:** Primer sequences used for real-time PCR.

Genes	Forward Primer (5’ -3’)	Reverse Primer (5’ -3’)
BMP4	TTGATACCTGAGACCGGGAAG	ACATCTGTAGAAGTGTCGCCTC
NOGGIN	GCCAGCACTATCTACACATCC	GCGTCTCGTTCAGATCCTTCTC
Col1	CGTCGGAGCAGACGGGAGTTT	CAGAGTTTGGAACTTACTGTC
SPARC	CTGCGTGTG AAGAAGATCCA	CATGTGGGTTCTGACTGGTG
FGF9	CCAGGACTAAACGGCACCAGAA	AATAAGAACCCACCGCATGAAAG

### 2.7 Statistical analyses

SPSS27.0 statistical software was used for statistical analysis. The normality test was performed on measurement data. Quantitative data were presented as mean ± Standard Error of Mean (SEM), and the data were analyzed by Independent-Samples T-Test. Differences were considered significant at P < 0.05.

## 3 Results

### 3.1 T1DM animal model

**Figure 1:**
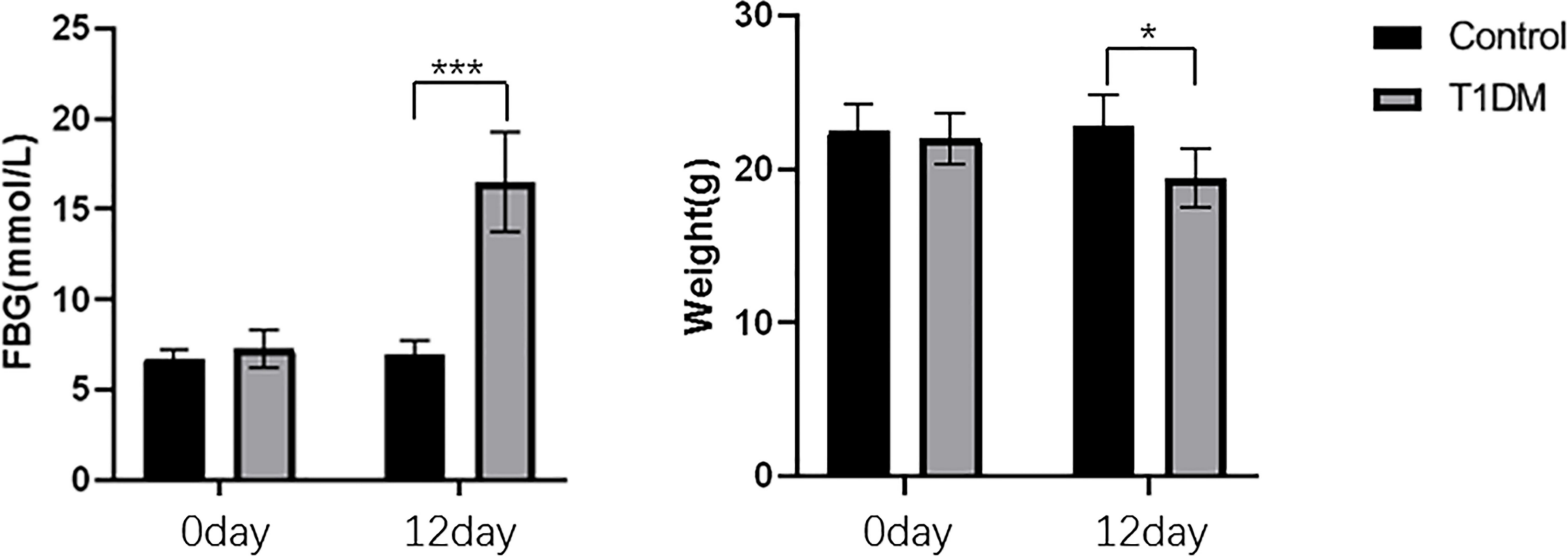
The changes of FBG and body weight in control and T1DM groups. Compared with the control group, the blood glucose was significantly increased, and the body weight was decreased in the T1DM group. *P < 0.05; ***P < 0.001.

A total of 46 mice were modeled, and all 30 mice in the control group survived. The body weight of mice in STZ-injected was significantly lower than that of the control mice(19.44 ± 1.92g vs. 22.76 ± 2.08g, *P* = 0.016). Mice in the STZ-injected group, the blood glucose levels were significantly higher than that of control mice(16.49 ± 2.75mmol/L vs. 6.94 ± 0.83 mmol/L, *P* < 0.001). The induction of the T1 T1DM model is characterized by a significant increase in fasting blood glucose and weight loss. These characteristics indicated that the T1DM model was successfully established (**Figure 1**).

### 3.2 Eruption rate

**Figure 2:**
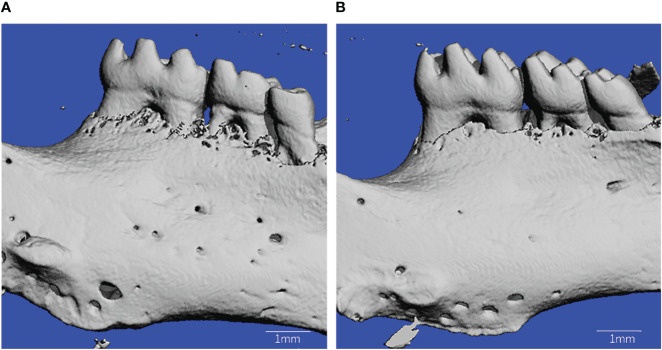
Microcomputed tomography reconstruction of mandibles in T1DM and control mice. **(A)** is from T1DM mice, and **(B)** is from control mice.

There was a significant amount of super-eruption on the unopposed side of both groups (**Figure 2**). The results demonstrated that the amount of tooth movement in the control group was significantly more than that in the T1DM group at 12 day after surgery(0.055 ± 0.014mm vs. 0.157 ± 0.017mm, *P* < 0.001).

### 3.3 Bone mineral density of the right mandible

**Figure 3:**
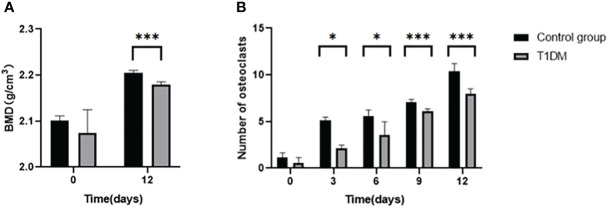
**(A)** is the bone mineral density(BMD) of the right mandible in control and T1DM groups on days 0 and 12 of the experiment.. After 12 days of tooth extraction, the bone mineral density in both groups increased during the axial tooth movement, and the increase of bone mineral density in the T1DM mice was significantly less than that in the control group. **(B)** is the number of right mandibular osteoclasts in both groups on days 0, 3, 6, 9, and 12 of the experiment. The number of osteoclasts in the two groups increased gradually. Compared with the control group, the number of osteoclasts in the T1DM mice was higher, which was particularly obvious at day 9 and day 12. *P < 0.05; ***P < 0.001.

After 12 days of tooth extraction, the density of the right mandible increased in both groups (**Figure 3A**). Compared with control mice, the T1DM group showed less increase in bone mineral density (2.180 ± 0.006 g/cm^3^ vs. 2.204 ± 0.0061 g/cm^3^, *P<0.001*).

### 3.4 Histological analysis

**Figure 4:**
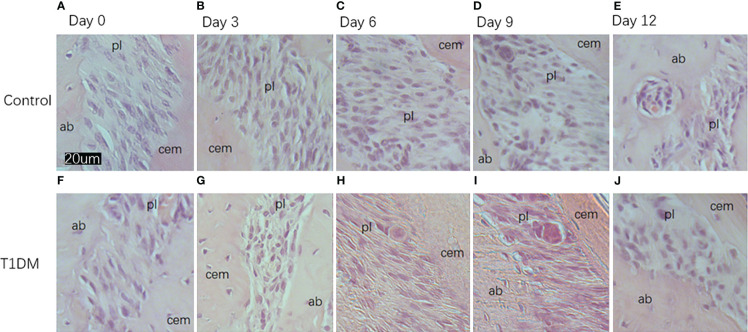
Histological appearance (HE staining)of the periodontium of mandibular molars in both groups on days 0, 3, 6, 9, and 12 of the experiment. **(A–E)** are from control mice, and **(F–J)** are from T1DM mice.3 to 12 days after the extraction of the surgery, the periodontal ligament of the corresponding mandibular molar showed irregular arrangement of cells. The cells in the periodontal ligament space, cementum, and alveolar bone surface gradually increased, and osteoclasts and osteoblasts could be observed in both groups of mice. In contrast, diabetic mice had fewer cellular components and a greater proportion of osteoclasts. cem, cementum; ab, alveolar bone; pl, periodontal ligament (×200).

**Figure 5:**
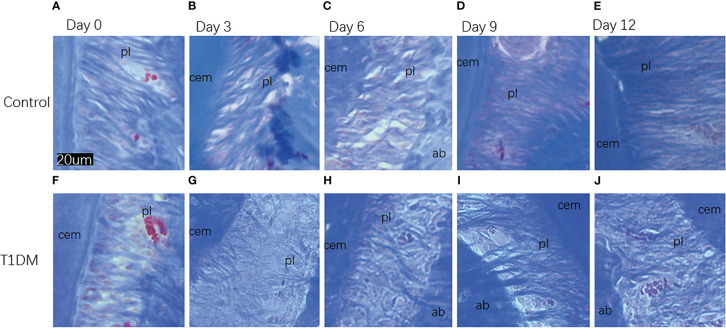
Histological appearance (Masson staining)of collagen fibers in the periodontal ligament in both groups on days 0, 3, 6, 9, and 12 of the experiment. **(A–E)** are from control mice, **(F–J)** are from T1DM mice.In both groups, at 3 and 6 days after the extraction of the right maxillary molar, the collagen fibers in the periodontal tissue of the mandibular molar began to arrange disorderly. At 9 and 12 days after the extraction, the collagen fibers became compact. Compared with the control group, the collagen fibers in the T1DM group were more sparse and disordered. cem, cementum; ab, alveolar bone; pl, periodontal ligament (×200).

**Figure 6:**
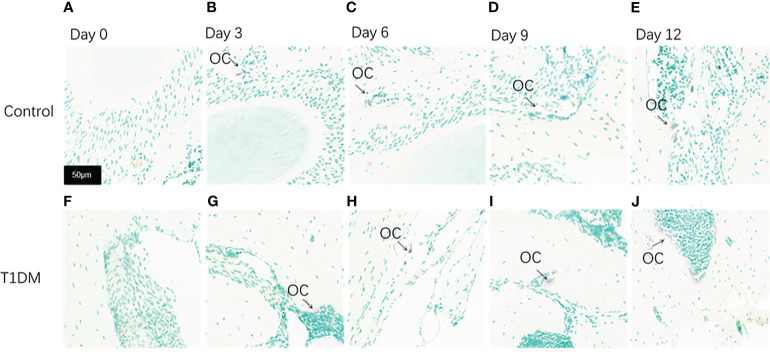
Tartrate-resistant acid phosphatase staining(TRAP staining) of the periodontium of mandibular molars in both groups on days 0, 3, 6, 9, and 12 of the experiment. **(A–E)** are from control mice, and **(F–J)** are from T1DM mice. 0 to 12 days after the extraction of the surgery, the number of osteoclasts increased gradually in both groups, however, the number of osteoclasts in the T1DM group was higher than that in the control group. The arrows indicate the site of positive osteoclast staining. OC, osteoclast (×200).

Histological analysis in all groups showed changes in the periodontal tissue structure of mice by HE staining (**Figure 4**) and Masson staining (**Figure 5**). TRAP staining (**Figure 6**) showed changes in the number of osteoclasts (**Figure 3B**). The histological trend of the T1DM group was consistent with that of the control group. In both groups of mice, the periodontal ligament was mainly composed of fibroblasts interspersed with collagen fibers and vascular and neural elements. Three days after the extraction of the right maxillary molars, cells were arranged irregularly, the periodontal collagen fiber bundles were arranged disorderly, and the cells on the surfaces of cementum and alveolar bone gradually increased. Collagen fibers also became more compact at 9~12days. In the control group, cells in the periodontal ligament were arranged in order, and the periodontal ligament fibers were orderly arranged. Compared with the control group, collagen arrangement was disordered and the number of osteoclasts (**Figure 3B**) was higher in the T1DM group (At days 3,6,9, and 12, *p*<0.05). There were fewer cellular components and sparse collagen fibers per unit area of alveolar bone in the T1DM mice.

### 3.5 RT−qPCR analysis

**Figure 7:**
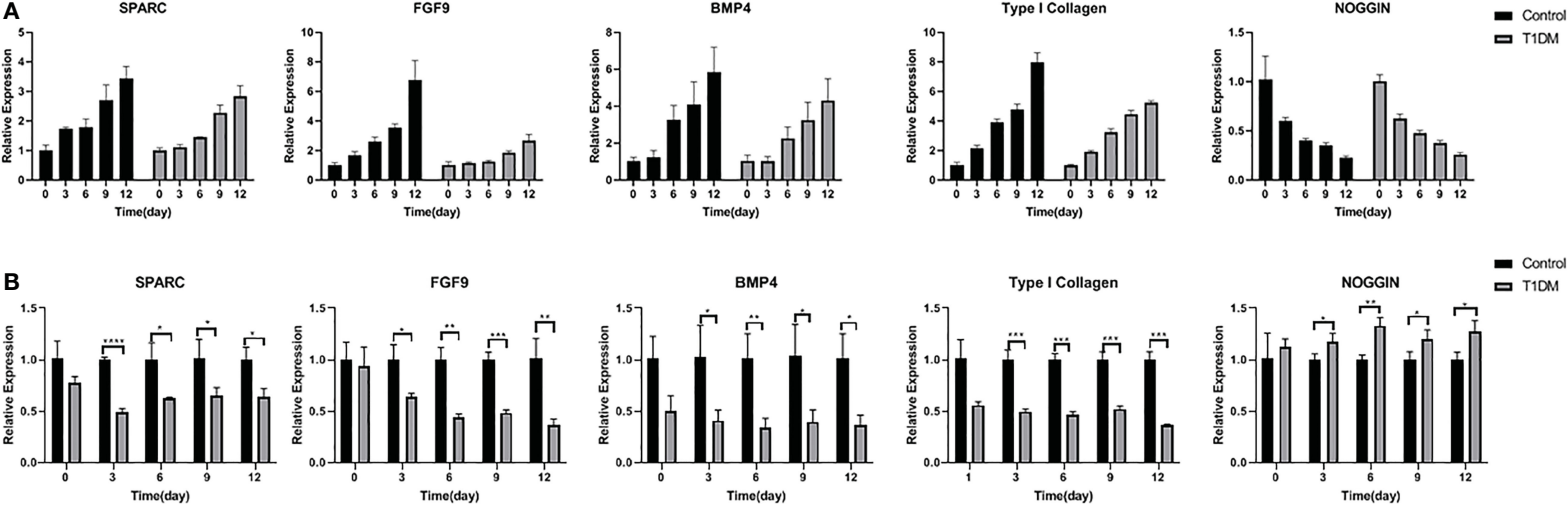
The relative expression of SPARC, FGF9, BMP4, Type I collagen and NOGGIN in control and T1DM group on days 0, 3, 6, 9, and 12 of the experiment. **(A)** The trend of relative expression levels in both groups at days o,3,6,9,12. Extraction of the opposing teeth resulted in a gradual increase in SPARC, FGF9, BMP4, and Type I collagen relative expression while the expression level of NOGGIN decreased, indicating that these osteogenesis-related factors were actively involved in bone remodeling during the axial tooth movement. **(B)** Comparison of the relative expression levels in two groups at different time points. The relative expression levels of SPARC, FGF9, BMP4, and Type I collagen were lower, and the NOGGIN expression was higher in T1DM mice compared with control mice, suggesting that osteogenesis was impaired in T1DM mice. *P < 0.05; **P < 0.01; ***P < 0.001; ****P < 0.0001.

The relative expression of SPARC, FGF9, BMP4, and type I collagen increased with the extension of tooth extraction time, while the relative expression of noggin decreased in the two groups. The relative expression of SPARC, FGF9, BMP4, and type I collagen in the T1DM mice were significantly lower than that in the control mice, and the relative expression of NOGGIN was higher (**Figure 7**)(At days 3,6,9, and 12, *p*<0.05).

## 4 Discussion

The axial tooth movement is the physiological mechanism of tooth eruption. The loss of antagonists results in varying degrees of axial tooth movement in the post-emergent phase of tooth eruption (20). Moreover, diabetes mellitus(DM) was a common disease which affected tooth movement. T1DM usually starts during adolescence, at a time of accelerated skeletal growth, and because the bone becomes compromised at a younger age, the adverse consequences are more severe relative to T2DM (21). In our study, the maxillary molars of mice were extracted to establish the model of the unopposed mouse molar, and we found that T1DM mice had a lower tendency of tooth super-eruption than normal mice(*P*<0.001). The periodontal ligament biological responses were inhibited, and the alveolar bone remodeling was weakened in type 1 diabetic T1DM mice.

Measurements of tooth movement were made through 3D reconstructed images and showed that the mandibular molars of T1DM mice in each group elongated after tooth extraction, and the elongation of type 1 diabetic T1DM mice was smaller. To find out whether external forces such as bite force or intrinsic genetic mechanisms worked for physiological tooth movement mechanisms, researchers developed the model of the unopposed mouse molar (22–24). Previous studies had established significant axial movement of first and second mandibular mouse molars following the complete extraction of antagonists (1). A study showed that diabetes significantly reduced orthodontic tooth movement (25). However, how type 1 diabetes T1DM affects axial tooth movement and its physiological mechanisms have not been studied.

In this study, bone mineral density BMD was measured during tooth elongation in both groups. The generation and functional regulation of osteoblasts and osteoclasts lead to changes in the bone mineral density BMD (26). The results showed that BMD increased in mandible of both groups, but the increment in diabetic group was smaller than that in control group. Previous studies had suggested that the decreased BMD in patients with type 1 diabetes T1DM was due to the retardation of osteoblast activity and the inhibition of bone remodeling (27). Uncontrolled blood glucose levels and insulin deficiency are thought to be the main causes of osteopenia in T1DM. Insulin can directly affect bone cells and may lead to low bone mineral density BMD in T1DM.

Our results showed that periodontal ligament collagen fibers and osteoclasts responded actively with increasing time, whereas T1DM mice had smaller responses and more osteoclast. Changes in the periodontal tissue were similar to those observed during physiological tooth development. It has been demonstrated in previous studies that axial tooth movement induced by unloading in mice was due to osteoclastic bone resorption on the distal aspect of the alveolar socket combined with alveolar bone and cementum formation on the mesial and apical parts of the alveolar socket (28). The changes of periodontal collagen and cells reflected the response of periodontal ligament during tooth movement with orthodontic treatment (29). Nevertheless, the detailed molecular mechanisms underlying the role of individual molecules in unloading-induced bone remodeling remain unclear.

The results of qPCR showed that the relative expression of extracellular matrix gene products SPARC and type I collagen in all mice increased with the extension of tooth extraction time, however, the relative expression of them in the T1DM group were significantly lower. Type I collagen is the main structural component of the PDL, which maintains periodontal health (30). In previous orthodontic experiments, the expression of type I collagen increased during tooth movement (31). SPARC (secreted protein, autogenous and rich in cysteine) could modulate osteoblasts and osteoclasts and was critical for normal bone remodeling. SPARC also could mediate the *in vitro* mineralization of type I collagen by binding firmly to type I collagen (32, 33). The regeneration capacity of PDL and bone formation were reduced in Sparc-null mice (34, 35). In previous studies, the relative expression of SPARC in the type 2 diabetes T2DM mice was higher than that in the control mice (36, 37), and this might be the result of SPARC promoting insulin resistance.

The relative expression of osteogenesis-related factors BMP4 and FGF9 were continuously increased while NOGGIN (Extracellular BMP antagonist, acts by binding BMP4 with high affinity, and as a consequence, blocks its biological effects) decreased. Compared with the control mice, the expression level of BMP4 and FGF9 was lower, while the NOGGIN expression level was higher in the T1DM mice. Bone morphogenetic proteins (BMPs) play a crucial role in regulating alveolar bone formation (38). BMP4 is identified as a bone-inducing factor, and injection of BMP4-transduced MSCs induced bone formation in mice (39). FGF9 has complex and essential roles in skeletal development and repair, inducing osteoblast proliferation and new bone formation (40). Some studies had demonstrated that Type 2 diabetes T2DM affected bone remodeling, leading to decreased bone regeneration. These effects could be reversed by the local application of FGF9 (41). Hyperglycemia is the main feature of T1DM. Hyperglycemia affects alveolar bone and periodontal ligament repair and reconstruction. Therefore, the unusual expression of these extracellular matrix gene products and osteogenesis-related factors in T1DM may be due to the impaired cells function and abnormal changes of bone protein matrix induced, leading to a chronic inflammatory state of bone (42).

However, this study had several limitations. First, our study only observed the expression changes of factors associated with tissue remodeling during tooth movement. The related genes should be knocked out in subsequent studies to determine whether the tissue remodeling ability is weakened or enhanced. Second, in this study, only the axial movement of mandibular teeth was observed, where the direction of gravity was opposite to the tooth elongation. The movement of maxillary teeth in the same direction of gravity should also be observed to research whether there was any influence in the next experiment. Finally, these experiments were currently limited to animal models, and further studies are needed to explore the data of axial tooth movement in type 1 diabetic T1DM patients.

In conclusion, the axial tooth movement was inhibited in type 1 diabetic T1DM mice. The mechanisms responsible for these pathological changes may be associated with enhanced periodontal ligament osteoclastogenic effects and reduced alveolar bone remodeling, based on the regulation of extracellular matrix and osteogenesis-related factors.

## Data availability statement

The raw data supporting the conclusions of this article will be made available by the authors, without undue reservation.

## Ethics statement

The animal study was reviewed and approved by Animal Ethics Committee of the Second Hospital of Shanxi Medical University.

## Author contributions

Guarantor of integrity of entire study, JN; study concepts/study design or data acquisition or data analysis/interpretation, all authors; manuscript drafting or manuscript revision for important intellectual content, all authors; agrees to ensure any questions related to the work are appropriately resolved, all authors; literature research, JZ, YX, and WN; clinical studies, WL; experimental studies, WL and DG; statistical analysis, JZ; and manuscript editing, all authors. All authors contributed to the article and approved the submitted version.
